# To boldly climb: behavioural and cognitive differences in migrating European glass eels

**DOI:** 10.1098/rsos.150665

**Published:** 2016-01-20

**Authors:** T. Podgorniak, S. Blanchet, E. De Oliveira, F. Daverat, F. Pierron

**Affiliations:** 1 Irstea Bordeaux, UR EABX, HYNES (Irstea—EDF R&D), 50 Avenue de Verdun, Cestas 33612 Cedex, France; 2 Station d’Écologie Expérimentale du CNRS à Moulis, USR 2936, 09200 Moulis, France; 3 CNRS, UPS, ENFA, Évolution et Diversité Biologique (EDB) UMR 5174, 118 Route de Narbonne, 31062 Toulouse, Cedex 9, France; 4 EDF R&D LNHE, HYNES (Irstea-EDF R&D), 6, quai Watier, Bat Q, Chatou 78400, France; 5 University Bordeaux, EPOC, UMR 5805, 33400 Talence, France; 6 CNRS, EPOC, UMR 5805, 33400 Talence, France

**Keywords:** eel, dams, behaviour, brain, cognition

## Abstract

European eel (*Anguilla anguilla*) is a catadromous fish species that received substantial attention as its population has markedly declined in the last three decades. The possible causes of this decline include habitat fragmentation factors such as dams and weirs. In some cases, these obstacles are equipped with fish friendly passage devices that may select young eels according to their climbing behaviour. We tested how individual climbing tendency was related to the event of fishway passage experienced in the field and classified fish climbing profiles as climbing ‘leaders’, ‘followers’, ‘finishers’ and ‘no climbers’. Moreover, we analysed the brain transcription level of genes related to neurogenesis and synaptic plasticity and compared it to climbing profiles. We found that fish from the upstream segments of an impounded river had a higher climbing propensity. Their behaviour was also more repeatable throughout the whole test than the obstacle-naive fish from the downstream segment. Moreover, we found that boldly climbing ‘leaders’ had lower levels of transcription of synapse-related genes than the climbing ‘followers’. These differences could be related to coping styles of fish, where proactive ‘leaders’ express a routine and risky behaviour, whereas reactive fish need an environmental assessment before exploratory behaviour. Our study showed that differences in climbing propensity exist in glass eels separated by water obstacles. Moreover, eels could adopt climbing different strategies according to the way they deal with environmental stress and to the cognitive abilities they possess.

## 1. Introduction

European eel (*Anguilla anguilla*) is a catadromous species with a high phenotypic plasticity [1,2], occupying a wide range of European inland and estuarine ecosystems. This species has a complex life cycle requiring two trans-Atlantic migrations. Firstly, leptocephali larvae migrate from the Sargasso Sea, their unique spawning ground, towards the European continental shelf [3]. Once arrived, they metamorphose into glass eels (i.e. post-larval stage) and many of them continue their migration to reach the upstream zones of inland waters, where they settle down for their juvenile growth phase. This stage can last from a few years to more than 20 years and ends with a second metamorphosis called silvering which prepares the future genitors (silver eels) for their transoceanic reproductive migration.

Owing to its strong population decline, the European eel is nowadays considered as ‘critically endangered’ [4], mainly because of overfishing, pollution, diseases and habitat fragmentation [5]. Habitat fragmentation is of growing concern today, as human-made water obstacles such as dams and weirs, built for river regulation and energy production are present on 80% of main European rivers [6]. Although eel possess a natural ability for climbing waterfalls, they are generally unable to perform such ascent on an artificial, dry concrete substrate, sometimes reaching up to 30 m in height. Increasing consciousness of the negative impact of such artificial obstacles on migratory fishes has led to the construction of fish friendly devices such as fishways and fish ladders. Fish passage devices are meant to facilitate fish migratory routes and their design improvements are subject of many ongoing studies [7,8]. However, while the efficiency of fish friendly devices is often assessed quantitatively [9], little is known about their impact on fish individual features and selectivity [10]. Indeed, in order to reach the upper tributaries, migrating eels need first to find the entrance of the pass and voluntarily express a willingness to climb, i.e. engage into the fishway and go out of the water. Secondly, they need to find the way through the fishway and be able to reach its upper zone, especially in case of long, steep and potentially energy demanding fish ladders. Thirdly, the event of climbing fish friendly devices, often performed under unusual environmental conditions, require from fish a different environmental perception, which could lead to changes in individual phenotypic traits, such as those related to neural activity [11]. In case of demographically self-sustaining populations, this selective impact of fish passes would reinforce or generate phenotypic differentiation within populations [12]. In the case of catadromous and panmictic eel species, fragmentation by weirs and dams could for instance generate phenotypically different eel population subsets below and above the obstacle.

Our study aimed at testing experimentally whether juvenile eels found in the upstream parts of an impounded river express a different climbing behaviour than those found in downstream areas. We hypothesized that the tendency to climb should be higher in fish from upstream areas because: (i) the behavioural selection has already operated on them: the upstream group of fish would contain only fish with innate climbing tendency, and because (ii) fish that have already experienced and succeeded in climbing across fish passes in the field would more likely climb an experimental fish pass device than the ‘untrained’ downstream fish. To test this hypothesis, we sampled juvenile eels from four different zones along an impounded river gradient and tested their climbing behaviour under standardized experimental conditions, i.e. on an experimental fish ladder. Moreover, as differences in the transcription level of genes related to cognitive function had already been detected among similar groups of fishes [11], and their long-term persistence was recently shown [10], we additionally measured the transcription level of four genes related to long lasting regulation of cognitive function [13,14] and compared it to experimentally measured climbing behaviour. Indeed, studies combining individual data on the current state of the fish (i.e. physiological analyses, behavioural tests, molecular profiles) have been recently proposed as a novel tool in research aimed at assessing the influences of hydropower barriers on fish populations [7]. We believe that combining these different approaches would help to better address the ecological and evolutionary consequences of habitat fragmentation on fish populations, notably by providing integrative responses of organisms to such stresses.

## 2. Material and methods

### 2.1 Sampling

**Figure 1.**
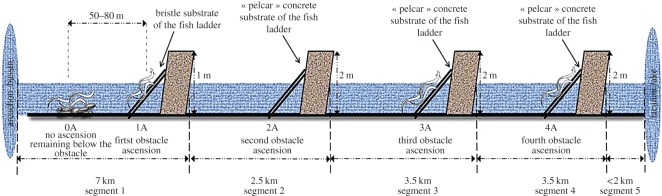
Sampling site characteristics. The height and type of the fishway in the first segment are different from the upstream segments. Fish from the group 0A and 1A are from the same segment, but caught when expressing different behaviour (remaining hidden in the substrate (0A) or ascending the fishway (1A)). Sampling size of the studied groups: 0A=11, 1A=9, 3A=15, 4A=14. Owing to the insufficient sampling size of the 2A group, fish from the second segment were not included in the study.

Eels were collected using electric fishing during the 16th and 17th of June 2014 under similar climatic and hydrological conditions in the Canal des Etangs, an artificial freshwater corridor in the southwestern part of France (44.75–44.95° N, 1.1–1.2° W). The aquatic corridor is linear and the water flow is homogeneous as it is controlled by a series of weirs (figure 1). Four successive low-distanced obstacles were built along the main channel, all equipped with a fish pass delimiting three successive river segments. The most downstream dam is equipped with a glass eel-specific pass, the three other are equipped with an eel pass (figure 1). A total of 49 individuals were sampled and used during the subsequent analyses. The number of animals per sampling site was distributed as follows (0A=11, 1A=9, 3A=15, 4A=14) according to their body size (between 75 and 85 mm) and health status (no externally visible pathogens). By sampling linear dammed sites, we ensure that certain fish have already expressed different climbing behaviour in the field (with expectedly no climbing event, and from one up to four climbing events, hereafter labelled, respectively, as 0A, 1A, 3A and 4A). In the two most downstream segments, individuals were sampled below the obstacle (0A), or caught while climbing the first fishway (1A). Fish from all the upstream segments (3A, 4A) were sampled directly on the fishway slope. Despite sampling efforts, the sampling size of the group of fish caught while climbing the second obstacle (2A group) was insufficient (six individuals of targeted body length), and was therefore not included in the study. All fish were brought alive to the laboratory for the behavioural test.

### 2.2 Behavioural test

**Figure 2.**
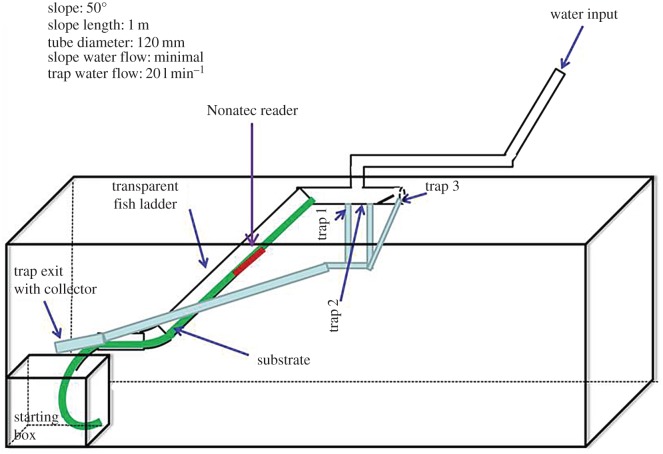
Schematic representation of the experimental fishway device.

After a prophylactic treatment (H_2_O_2_, 250 ppm, 60 min), eels were individually marked by inserting with a sterile needle (0.23×4 mm) a 6 mm long RFID device (Nonatec, Lutronics) in the peritoneal cavity. Tagging, as well as body weight and length measurements were undertaken under fish anaesthesia (0.3 ml of eugenol, dissolved 1 : 10 in 95% ethanol and diluted in 10 l of water). After one week recovery in the same controlled conditions of light, food, temperature and water flow (*T*=22°C, pH=6, 12 L : 12 cycle, fed with *Chironomidae* ad libitum), fish were placed into an experimental fishway (figure 2) and their climbing behaviour in group was observed during each of four consecutive experimental trials. Three behaviour variables were taken into account for each eel: the total number of ascensions, the mean number of ascensions *per* trial and the mean rank score of their first ascension. The mean rank score represents how fast each individual climbed for the first time the experimental device during each of the four trials. For each trial independently, the fish received 1 point of the rank score if its first ascension was among the fastest fish, i.e. if it climbed in front position within the first 33% of the climbing fish. All the fish whose first climb was situated within 33 and 66% of all the climbing fish received 2 points of the rank score and all the remaining climbers of the test received 3 points. If the fish did not climb during the trial, it received 4 points of the rank score. Thus, the fish that never climbed during all the four trials, received 16 points of the rank score, and their mean rank score was 4. On the other extreme, the individuals that always climbed in the first third part of the fish during all four trials received 4 points, with the mean rank score of 1. The mean rank score allows the assigning of each fish to one of four classes of ‘peloton’: ‘leaders’, ‘followers’, ‘finishers’ ands ‘no climbers’. A fish became a ‘leader’ if its mean rank score was less than 2, a ‘follower’ had the mean rank score within 2 and less than 3, and the ‘finisher’ within 3 and less than 4. The ‘no climbers’ class had a mean rank score of 4.

The experimental fishway was specifically built for behavioural analysis, i.e. the climbing behaviour test, which consisted of four separate 300 min trials. At the beginning of each trial, fish were placed in the starting box connected to a transparent tube inclined at 50° (figure 2). The transparent tube was equipped with plastic grass-like substrate, similar to the substrate used for glass eel ladders. The upper part of the transparent tube was connected to a horizontal opaque tube with holes. The water was flowing through the tube (water flow on the slope was minimal) to induce fish climbing until the upper zone, where fish were caught into a fishnet-like collector. Turbulences in the upper zone of the tube were high, leading to the drop of fish into the trap directly connected to the starting box. During each trial, each climbing fish is automatically identified using Nonatec^*TM*^ Arm Reader (Lutronic) connected in the middle of the climbing slope, before it gets trapped and falls back into the starting box. At the end of each trail, the water flow was increased to ensure that the climbing fish fall down into the trap while preventing the others from climbing. The time lapse between each trial was 24 h. The whole experimental device was cleaned after each trial, to avoid interference with the odours from the previous test [15].

At the end of the experiment, body weight and length were measured, the monthly growth rates and body condition factors [16] were calculated, then fish were sacrificed by severing the medulla oblongata. The whole brain was extracted and stored in RNALater^®^ buffer (Qiagen) for gene transcription analyses.

### 2.3 Gene transcription analysis

Four genes were chosen to specifically target a potentially long-term cognitive function in the brain (table 1). Four genes are associated with synaptic plasticity or its long-term effects on neural activity (long-term potentiation, LTP), i.e. activity-dependent strengthening of synapses [17,18], and some were also associated with social behaviour, coping styles, memory or learning [10,19–21].

**Table 1.** Primer pairs used for RT-qPCR analyses (*a*=Forward, *b*=Reverse). A total of four genes were chosen for brain analysis. *β*-*actin* was chosen as the reference gene.

short name	primer sequence	function	BLAST hit name	species	*E*-value
*camk2g*	GACGGAACTAAGGGGTCCTCa	memory, synapse, LTP	XM_006630861.1	*Lepistoseus oculatus*	0
	AGGTCAACCCAGGATCACAGb		GI:573886134		
*jun*	GATTCGACGTTCACGGTTTTa	memory, synapse, LTP	JN257262.1 GI: 357595814	*Carassius auratus*	0.0
	TGTGGTTGACGCATTTCATTb				
*bdnf*	GGTCATCACTCTTCCCACCTa	learning behaviour, IEG	XM_010793251.1	*Notothenia coniceps*	7.0×10^−66^
	AACCATGCAATTTCCACCATb		GI: 736296403		
*egr-1*	ACCTACTCCAGTGCCAGCTCa	learning behaviour, IEG	JN230914.1 GI: 389566557	*Conger conger*	0
	GAACAGGTAGTCGGGGATCAb				
*β*-*actin*	CAGCCTTCCTTCCTGGGTa	housekeeping gene	DQ286836.1 GI: 82798415	*Anguilla anguilla*	0
	AGTATTTGCGCTCGGGTGb				

For each gene, specific primer pairs were designed using the Primer3Plus software [22] and were purchased from Sigma Aldrich. All primer pairs were reported in table 1. RNA extraction and qPCR analyses were performed as previously described [11]. Relative quantification of each gene transcription level was normalized according to the *β*-*actin* gene transcription. Hence, during our experiment, total RNAs were quantified and a same quantity was used for reverse-transcription. During the subsequent qPCR amplifications, the output cycle corresponding to *β*-*actin* was examined. This output was always obtained around the same output cycle and no significant variations were observed among conditions, demonstrating the relevance of the *β*-*actin* as the reference gene in our conditions.

### 2.4 Data treatment and statistical analyses

Comparisons of body length, body weight and body condition factor among fish groups from different river sections (0A, 1A, 3A, 4A) or from different classes of peloton were performed after testing the assumptions of normality (Shapiro–Wilk test) and homoscedascity (Bartlett test) of the error terms. When these two assumptions were met, ANOVA analysis was used. When they were not met, a non-parametric Kruskal–Wallis test was applied. If significant effects were detected, a Tukey HSD or Wilcoxon tests (respectively) were used to determine whether means or medians significantly varied between pairs of samples.

Concerning the behavioural test, the total count of fish pass ascension was compared among groups using generalized linear models (GLMs) with a Poisson error-term distribution, whereas the frequency of ‘peloton’ classes was compared among groups using a *χ*^2^-test for frequency data (package MASS). The transcription level of the four genes was compared among the classes of ‘peloton’ (Kruskall–Wallis and Wilcoxon test, as the normality assumptions were not met). In addition, the repeatability of fish behavioural traits (number of ascensions *per* trial, rank score of first ascension *per* trial) was tested using intraclass correlation coefficient (ICC, package ICC) [23]. The ICC describes how strongly the units of the same group resemble each other and is based on the variance within versus among groups [23].

For all the statistical results, a probability of *p*<0.05 was considered significant.

## 3. Results

### 3.1 Body length, weight and condition

No differences in fish initial (80±7.6 mm; $\chi_{3}^{2}=1.18$, *p*-value =0.76), and final body lengths (80.6±7.4 mm; $\chi_{3}^{2}=1.13$, *p*-value =0.77), or weights (initial: 517±194 mg; $\chi_{3}^{2}=0.36$, *p*-value =0.95; final: 554±209 mg, $\chi_{3}^{2}=1.32$, *p*-value =0.72) of fish were observed among groups.

### 3.2 Sampling site of fish

**Figure 3.**
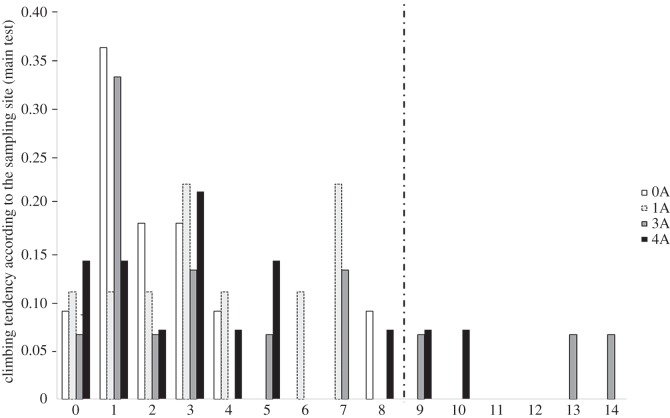
Proportion of fish from different sampling sites and expressing different climbing tendency (i.e. total number of ascensions). Sample sizes of the fish groups coming from different sampling sites: 0A=11, 1A=9, 3A=15, 4A=14.

Significant differences in climbing tendency were detected between 0A and 3A field groups (GLM, Poisson distribution, d.f. =48, *z*-value =2.82, *p*-value =0.005) and between 0A and 4A field groups (GLM, Poisson distribution, d.f. =48, *z*-value =2.05, *p*-value =0.04). Indeed, fish that climbed more than eight times in total during the whole test originated only from the most upstream groups, i.e. 3A and 4A (figure 3).

We found moderate to high significant levels of repeatability values for individuals from 3A and 4A groups and for the two behavioural traits, i.e. the number of ascension *per* trial as well as the rank score *per* trial (table 2). In other words, fish from the 3A and 4A groups kept a similar position in climbing groups and performed a similar number of ascensions throughout all the trials. Contrastingly, in the groups containing obstacle naive fish (0A and 1A to a lesser extent) repeatability values were low and not significant for any of the two traits (table 2, the 95% CI included 0 for the two traits).

**Table 2.** Repeatability of two behavioural variables, number of climbs *per* trial and rank score of the first climb *per* trial with respect to the sampling site of fish. (The 95% confidence intervals (CIs) are given for each value. The repeatability values in bold are statistically significant (lower CI > 0). Sampling size of groups: 0A = 11, 1A = 9, 3A = 15, 4A = 14.)

ICC						
	climb count per trial			rank score per trial		
origin	(lower CI)	value	(upper CI)	(lower CI)	value	(upper CI)
0A	(–0.054)	0.192	(0.575)	(–0.170)	0.008	(0.374)
1A	(–0.102)	0.149	(0.586)	(–0.116)	0.127	(0.564)
3A	(0.254)	**0.508**	(0.759)	(0.244)	**0.499**	(0.753)
4A	(0.153)	**0.415**	(0.707)	(0.023)	**0.265**	(0.590)

### 3.3 Individual behaviour of fish

The group of fish labelled as ‘leaders’ (fish that climbed among the first third part of the fish during each trial) expressed the highest number of ascensions during the whole test. This group had also the highest (although not significant) repeatability value in climbing tendency. At the opposite end, the group of ‘finishers’ had the lowest repeatability value in climbing tendency and the lowest average number of ascensions during the whole test (table 3).

**Table 3.** Mean number of total climbs (i.e. climbing tendency), mean rank score, repeatability of climbing behaviour and number of individuals corresponding to the different classes of peloton. (Standard errors (s.d.) are provided for the first two variables, whereas the 95% CIs are given for the third one (repeatability).)

peloton variable mean (s.d./CI)	leader	follower	finisher	no climber
climb count *per* trial	8.5 (2.6)	6.1 (3.1)	1.8 (0.96)	0
rank score	1.5 (0.27)	2.4 (0.26)	3.4 (0.29)	4 (0)
repeatability of first climb *per* trial	(–0.09) 0.21 (0.70)	(–0.10) 0.08 (0.40)	(–0.22) –0.15 (–0.01)	(0) 4 (0)
group size	6	14	24	5

### 3.4 Distribution of individual behaviours according to the origin of fish

**Figure 4.**
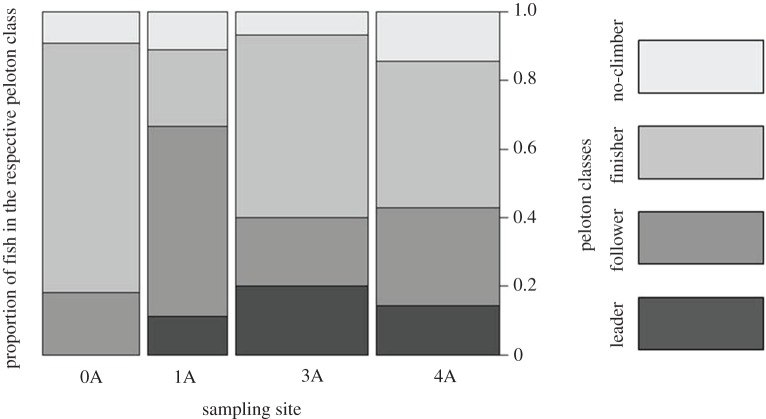
Proportion of fish of different ‘peloton’ classes (mean rank score during their first climb on the fishway) according to their sampling site. Four classes (leaders, followers, finishers, no climbers) and four sampling sites (0A, 1A, 3A, 4A) were compared. Size of peloton classes: leaders =6, followers=14, finishers =24, no climbers=5. Size of sampling site groups: 0A=11, 1A=9, 3A=15, 4A=14.

The overall proportions of fish of the different classes of variable peloton did not vary significantly among the sampling sites ($\chi_{9}^{2}=8.41$, *p*-value =0.49). Interestingly, we observed that the 0A group was the only group that did not include any ‘leader’ fish (figure 4).

### 3.5 Gene transcription levels

No differences were detected in gene transcription levels among sampling site groups.

**Figure 5.**
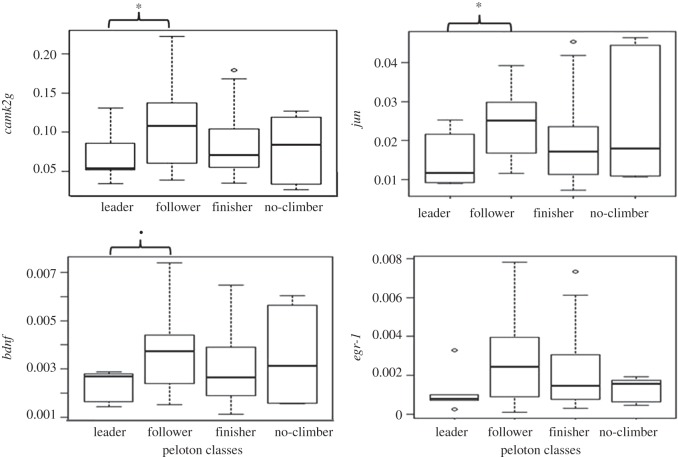
Mean gene transcription levels (arb. units) of fish with different climbing behaviour (i.e. peloton classes: leaders, followers, finishers and no climbers). Sampling size of groups: leader =6, follower=14, finisher =24, no climber=5. The significance level of the *p*-value is indicated with an asterisk (0.01<*<0.05<0.1).

Significant differences were observed between the peloton group of leaders and followers in the gene transcription level of *camk2g* (*W*=16, *p*-value =0.04), *jun* (*W*=14, *p*-value =0.03) and *bdnf* (*W*=17, *p*-value =0.05). Significant higher transcription levels were observed in followers in comparison to leaders (figure 5). Finishers presented intermediate values. Despite a similar pattern detected for the gene *egr-1* (increased level of transcription in followers compared with leaders), no significant difference was observed (*W*=19, *p*-value =0.08).

## 4. Discussion

This study focused on two behavioural variables of climbing. First, we accounted for the total number of ascensions, which could be interpreted as a result of climbing tendency and capacity. Second, we studied the order in which fish climbed for the first time during each trial, which could refer to behavioural traits such as the propensity to explore the new habitat, and that are often associated with bold personality types [24,25]. Moreover, the repeatability of this climbing order gave insight on how consistently the fish behave within the same sampling site group or within the group of similar mean ranking score.

Differences in climbing tendency were significant among sampling site groups, and the fish displaying extreme behaviour (more than eight climbing events) originated from the most upstream groups (3A and 4A). Moreover, we found that the repeatability in climbing tendency was significant and higher in the two upstream groups (3A and 4A), whereas it was not significant in the two most downstream sites (0A and 1A) harbouring fish with low or even no climbing experience. In addition, fish from the most upstream zones had the highest repeatability in climbing order, i.e. behaved in a more consistent way than the fish from the downstream zones. These behavioural extremes could be somehow associated with the events of obstacle ascension experienced in the field. Although it is still difficult to tease apart whether the association between fish distribution and climbing behaviour is due to selection or habituation, we undoubtedly demonstrate for the first time, to our knowledge, that fragmentation induced by humans lead to a strong downstream to upstream differentiation in climbing behaviour in eels. In order to deepen these results and get a new insight, we then investigated more precisely the rank of passage of individuals. The first fish climbing the pass device are of particular value. Indeed, these individuals needed to spatially explore the new, potentially hazardous zones and negotiate the passage without any social or chemical prior information. Their behaviour could involuntarily give insights on how risky the upstream zone is (by releasing alarm cues such as cortisol hormones into the water [26,27]) and lead the other fish of the group to follow, this being of particular importance in shaping the dynamics of the whole group. We thus classified the individuals according to their rank score, without taking into account their origin. We identified four classes called ‘leaders’, ‘followers’, ‘finishers’ and ‘no climbers’. We found that the group of leaders not only expressed the highest number of ascensions during the whole test but also presented the highest repeatability in climbing tendency. We found that ‘leaders’ were absent in the obstacle-naive group (0A). In other words, the group of the boldly climbing and motivated fish was found only in the upstream zones of the river axis. This is consistent with the fact that fish from the upstream zones (3A and 4A) had not only the highest climbing propensity but also a more consistent behaviour than fish from the obstacle-naive group. Fish with low behavioural flexibility [28–30] and with lower aversion for risk-taking decisions are usually referred as proactive [31], in contrast to fish expressing a reactive coping style [19,32,33]. Reactive coping style is characterized by higher environmental appraisal, information processing and flexible behaviour highly dependent on stress and social cues, in opposition to proactive coping style, where individuals behave in a stereotypic, rather inflexible behaviour [28,30,34,35]. It has been shown that many species cope with socially or physically stressful situations with a simple dichotomy of heritable strategies, i.e. they adopt a proactive or reactive response to the environment [31,36–38]. In our study, fish labelled as leaders could be associated with proactive coping style, as they perform a risky behaviour in climbing a new area without any social cues, guided only by their rheotactic behaviour. Fish labelled as followers would benefit from these cues and thus would be able to process the environmental information to assess the situation before engaging into the fishway. They have developed the ability for cognitive processes to a larger extent than the proactive leaders. In support of this hypothesis, the transcription level of several genes was significantly higher in the brain of followers in comparison to leaders. These genes are associated with neural activity, neurogenesis and brain plasticity. Such results are in agreement with the literature where typically routine dependent and inflexible behaviour in proactive individuals is associated with low transcription of neurogenesis-related genes [19]. Among differentially regulated genes, *camk2g* is a one of type 2 calcium/calmodulin-dependent protein kinases, Ca^2+^-activated enzymes are associated with experience-dependent neural plasticity and behavioural memory as they control LTP [17,39], a molecular process of strengthening active synapses induced during learning and memory formation [40]. Similarly, C-JUN is a protein encoded by the *jun* gene and is a part of the activator protein (AP-1) early response transcription factor. *jun* is closely associated with synaptic plasticity and is involved in memory formation and learning [41,42]. Finally, the third gene *bdnf* encodes for a brain-derived neurothropic factor involved in neurogenesis and synaptic plasticity, both related to learning and memory [43–45]. The gene *bdnf* together with *egr-1* (early growth response protein 1) are a part of the Immediate Early Genes (IEG) family. They represent a standing response mechanism that is rapidly activated at the transcription level in response to stimuli, before any new proteins are synthesized. Experimental study showed an overexpression of *bdnf* in rainbow trouts that learned to escape the dominant individuals [46]. In an experimental study on African cichlid, a high activity of *egr-1* was shown in the profile of ‘learners’ when compared with no learning fish [21]. In our study, *egr-1* did not show significant differences although the patterns detected between leaders and followers were similar to *bdnf* (higher transcription level in followers), thus reinforcing our hypothesis on differences in cognitive abilities between leaders and followers. In potentially challenging or stressful situations, a correct appraisal of the situation, learning and memory would allow the shaping of an adaptive behavioural response, where neurogenesis and synaptic modifications would play a role in underlying behavioural plasticity [19]. Here, cognitive processes can be particularly important in individuals trying to adjust their behaviour in response to environmental variations [47,48]. However, mechanisms implied in cognition may provide a substrate to enhanced behavioural flexibility, but higher influence of environment on behaviour can also involve a higher responsiveness to stressful conditions [30].

Our results can have an important ecological meaning when situated in the sampling context. As the sampling was performed at the end of the migration period, all the bold climbers, the ‘leaders’, have already climbed or were engaged at least into the first field obstacle fishway and thus were caught further upstream. In order to pass the first water obstacle, fish remaining downstream waited a new ‘migration window’, i.e. the arrival of new group of migrants perhaps containing some new boldly climbing individuals, behavioural variants hitherto called the climbing ‘leaders’. Indeed, an experimental study on zebrafish showed that the whole group can adopt a bolder behaviour in presence of new bold individuals [49]. The migration waves of glass eels through an impounded axis could be shaped by the arrival of the fish willing to explore fish passes without any social cues and possibility of risk-assessment of such behaviour.

Boldly climbing leaders could be followed by reactive individuals, which in turn would be followed by the main part of the resting fish. These last fish are not necessarily reactive as they could simply adopt the most common behaviour of the group (around two-thirds of the fish have climbed, with some individuals climbing several times per trial). This positive frequency dependence is a phenomenon known as social conformity or ‘copy the majority’ strategy [50], although empirical evidence in fish is still scarce [49].

In conclusion, our study suggests that different climbing behaviours exist in European glass eel. Those behaviours could be associated with cognitive performance, as well as coping styles (proactive–reactive responses) the fish adopt to deal with the environment. These results are of important ecological relevance. Boldly climbing fish are more likely found in the upstream zones of the impounded river axis. This implies that water obstacles act as selective filters on behavioural strategies of fish. If these behaviours have a (epi) genetic basis, considering that the escapement success of upstream silver eels is lower than downstream animals (e.g. due to increased mortality associated with fish growing in upstream river and turbines of hydropower stations [51,52]), we could suggest a switch in (epi) genetic polymorphism in the offspring, owing to, for example, reduced contribution of bold fish to the semelparous event of reproduction.

Finally, as the aquatic obstacles are implemented in the upstream migratory routes of glass eels to such a level that climbing is a part of migration phenomenon, we believe investigating how the dynamics of the whole group of climbers is shaped by the presence of the leaders could help explain why well-known abiotic factors (weather, temperature, water discharge) sometimes fail in predicting the migration waves of glass eels.

## Supplementary Material

Data_eels

## Data accessibility

The dataset supporting this article was uploaded as part of the electronic supplementary material.
